# Retraction: Spatio-Temporal analysis of exports of cultural products and their affecting factors for spatial distribution

**DOI:** 10.1371/journal.pone.0308629

**Published:** 2024-08-06

**Authors:** The PLOS ONE Editors

The *PLOS ONE* Editors retract this article [1] because it was identified as one of a series of submissions for which we have concerns about the article’s adherence to *PLOS ONE*’s Ethical Publishing Practice polices, including but not restricted to concerns about the authorship and the integrity of the article’s peer review. We regret that the issues were not identified prior to the article’s publication.

The author either did not respond directly or could not be reached.
